# Monosodium glutamate-induced oxidative kidney damage and possible mechanisms: a mini-review

**DOI:** 10.1186/s12929-015-0192-5

**Published:** 2015-10-22

**Authors:** Amod Sharma

**Affiliations:** Department of Physiology, Siriraj Hospital, Mahidol University, Bangkok, 10700 Thailand; Department of Biochemistry, Faculty of Medicine, Khon Kaen University, Khon Kaen, 40002 Thailand

**Keywords:** Monosodium glutamate, Kidney, Oxidative stress, Glutamate receptors, α-Ketoglutarate dehydrogenase

## Abstract

Animal studies suggest that chronic monosodium glutamate (MSG) intake induces kidney damage by oxidative stress. However, the underlying mechanisms are still unclear, despite the growing evidence and consensus that α-ketoglutarate dehydrogenase, glutamate receptors and cystine-glutamate antiporter play an important role in up-regulation of oxidative stress in MSG-induced renal toxicity. This review summaries evidence from studies into MSG-induced renal oxidative damage, possible mechanisms and their importance from a toxicological viewpoint.

## Introduction

Monosodium glutamate (MSG) is a commonly-used additive in processed food and Asian cuisine to increase palatability. However, several studies in animals have shown that MSG is toxic to the various organs such as the liver, brain, thymus, and kidneys [1–3]. Published data indicate that renal fibrosis is associated with the chronic consumption of MSG [4] and oxidative stress is the main cause of kidney injury [5].

Oxidative stress is caused by the excessive production or a decreased elimination of free radicals in cells, the majority of which are oxygen radicals and other reactive oxygen species (ROS) [6]. Nutrition metabolism and several extracellular and intracellular factors such as hormones, cytokines, and detoxification processes contribute to the oxidative stress [7–9]. Therefore, excessive renal metabolism of glutamate as in chronic MSG intake can be a source of ROS. Decreased levels of major anti-oxidant enzymes and increased lipid peroxidation have been demonstrated in the kidneys of chronic MSG-exposed rats [10, 11]. Also, high doses of glutamate have been shown to induce significant toxicity in renal culture cells [12].

The abundance of long-chain polyunsaturated fatty acids in the composition of renal lipids makes kidney susceptible to damage by ROS [13].This makes kidney tissues prone to damage by different mechanisms such as the promotion of lipid peroxidation, protein modification, and DNA damage, leading to cell death [14–16]. Accordingly, the involvement of ROS has been reported in glomerular, tubular, and tubulo-interstitial alterations [17, 18].

A host of studies have explained glutamate-induced oxidative damage in tissues like brain or neurons, where α-ketoglutarate dehydrogenase, glutamate receptors and cystine-glutamate antiporter are the vital players [19–21].These molecules can contribute to the oxidative stress through, different mechanisms but little is known about their involvement in MSG-induced renal oxidative stress. The increased level of α-ketoglutarate dehydrogenase has been found in the kidney of MSG-fed rats [5] and accordingly, a strong consensus is being developed against α-ketoglutarate dehydrogenase, glutamate receptors, and cystine-glutamate antiporter for their potential role in the MSG-related renal oxidative stress. The purpose of this short review is to outline MSG-induced oxidative kidney damage and possible mechanisms.

## Review

###  MSG-induced kidney damage

The association between dietary factors, including MSG and the risk of kidney disease, has been hypothesized in numerous studies. The kidneys are highly sensitive to ischemia, toxic insults, and other chemicals. As such, processes leading to direct or indirect disturbances of renal cell energy metabolism will result in cell injury and acute renal insufficiency [22].

**Fig. 1:**
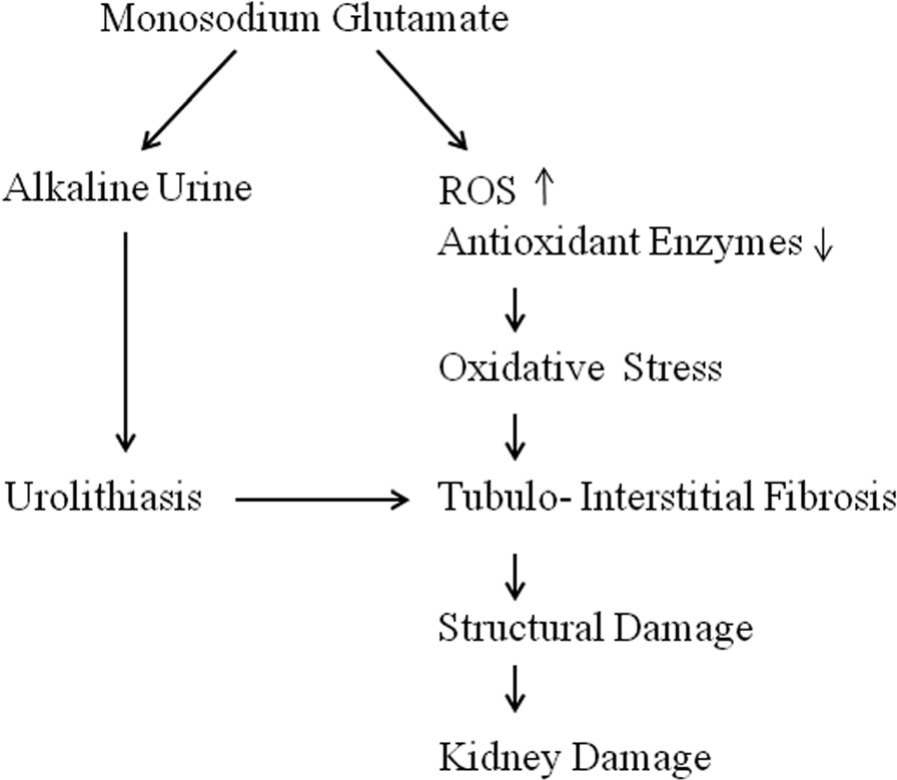
An outline of chronic MSG-induced renal alterations in the kidney. Alkaline urine and oxidative stress due to chronic MSG intake may damage the kidneys by unknown mechanisms. Urolithiasis can also contribute to the interstitial fibrosis by producing inflammatory cytokines and ROS

A summary of chronic MSG-induced renal alterations is illustrated in Fig. 1. MSG can induce changes in the renal cytoarchitecture, increase glomerular hyper-cellularity, infiltration of inflammatory cells in the renal cortex, edema of tubular cells, and eventually degeneration of renal tubules [10, 11, 23]. Although infiltration of inflammatory cells points towards a pathology, the exact pathophysiology is not fully understood. Cellular dysfunction is considered as an important cause of the subsequent development of most of the morphological alteration, regardless of the toxic principle acting upon the kidney. Therefore, ultra-structural examination of the kidney in experimental models with chronic MSG treatment could contribute to a better understanding of the mechanism of derangements during renal injury.

Experimental evidence of renal damage mediated by chronic MSG intake will be discussed further under oxidative stress, and urolithiasis, and interstitial fibrosis.

### Oxidative stress

The formation of ROS in the kidney exposed to MSG was seen as a major contributor to their nephrotoxic effects leading to cellular and functional damage [24]. MSG supplementation either by injection or oral intake has been shown to alter renal antioxidant system markers, including lipid peroxidation byproducts and kidney function in rats [10, 24]. Paul et al. (2012) found reduced activities of superoxide dismutase, catalase, glutathione-S-transferase and glutathione (GSH) in the kidney after MSG administration [10]. They also reported that markers for lipid peroxidation such as malondialdehyde (MDA) and conjugated dienes were increased in MSG treated renal tissue. It is possible that MSG leads to the excessive production of free radicals and endogenous antioxidants are insufficient to meet the demand. The up-regulation of heat shock cognate 70, an indicator of oxidative stress, and the down-regulation of glutathione-S-transferase in MSG-treated kidneys further strengthens the findings [5]. Moreover, some studies have found the ameliorating effect of vitamin C, E, and quercetin on MSG-treated kidneys [2, 10]. The mechanism whereby these antioxidants exert such effects is yet to be fully elucidated. However, these antioxidants do seem to play a key role against renal inflammatory responses through a diminution of the activity of inflammatory enzymes [25] and cytokines secretion, or by inhibiting the activity of NF-ĸB [26, 27].

Furthermore, studies using thiol antioxidants such as N-acetylcysteine (NAC) and lipoic acid have demonstrated therapeutic protection against glutamate-induced neurotoxicity [28, 29]. Although there is no experimental evidence available supporting the protective effect of these molecules in MSG-induced renal oxidative toxicity, NAC has been shown to reduce kidney MDA levels in a diabetic mouse model [30]. In cultured human proximal tubular epithelial cells, NAC reduced lipid peroxidation and maintained mitochondrial membrane potential, thereby preventing apoptosis following hydrogen peroxide administration [31]. Also, lipoic acid has been effective in protecting kidneys from oxidative stress and mitochondrial dysfunction [32]. In a different context, the ameliorating effect of selenium on MSG-induced testicular oxidative toxicity has been demonstrated [33]. These important findings add further prospective to the therapy of MSG-induced renal oxidative stress using antioxidants.

### Urolithiasis and interstitial fibrosis

Obstructive nephropathy due to chronic dietary MSG has been reported in adult rats probably due to alkaline urine and decreased levels of stone inhibitors such as magnesium and citrate in the urine [4]. The mechanism behind MSG-caused urine alkalization is still unknown but this effect was first reported by de Groot et al. (1988) [34]. It is likely that MSG-treated animals may generate higher catabolic products of glutamate in kidney cells and its carbon skeleton is converted into carbon dioxide and then to bicarbonate anions [35, 36]. The generated bicarbonates are then absorbed back into the blood circulation and ultimately to the kidneys for excretion of the extra-alkali, resulting in alkaline urine [37, 38]. Alkaline urine can influence the kidneys capacity in terms of secreting or reabsorbing metabolites that can contribute to stone formation, whereas inhibitors of stone formation play a major role in natural defense. An elevated ion activity product of calcium phosphate in the alkaline urine of MSG-fed mice indicates the risk of calcium-phosphate stone formation [4].

Furthermore, ROS can cause damage to the cells leading to cell death and formation of membrane-bound vesicles which support crystal nucleation [39, 40]. With this background, hydronephrosis with major changes such as fibrosis in the tubulo-interstitial compartment has been reported in MSG-treated rat kidneys by Sharma et al. (2013) [4]. It is important to note here that 2/10 of MSG-treated animals demonstrated a presence of hydronephrosis and 3/10 with renal stones in the study. However, all of the MSG treated rats had significantly high levels of renal fibrosis compared to the controls, suggesting the fibrotic effect of MSG, not merely renal obstruction. It is difficult to explain these distinct findings among the MSG-treated animals but the individual factors could have played a role. In a different experiment, our group was unable to notice the altered renal function or stones in rats at 1 month, 3 months, and 6 months of MSG treatment (unpublished data). However, altered kidney function and pathology but not the renal stones were reported by Paul et al. (2012) after 6 months of oral MSG treatment with higher dose. This indicates that the dose and duration of MSG exposure are vital for its nephrotoxic effects including stones and obstruction.

The mechanical disturbance resulting from complete ureteral obstruction causes tubular injury, resulting in a pro-inflammatory cytokines and tubulo-interstitial fibrosis [41]. Accordingly, in an experiment with a ureteral obstructed rat model, the investigators found increased 4-hydroxynoneal (4-HNE) stain for ROS products in the renal tubulo-interstitial compartment [42]. It can therefore be surmised that urolithiasis and oxidative stress due to MSG can cause fibrosis in the kidney, as ROS can induce the transformation of fibroblasts to myofibroblast [43]. Tubular interstitial fibrosis is highly associated with the progress of renal diseases [44].

### MSG-induced ROS generation in kidney

**Fig. 2:**
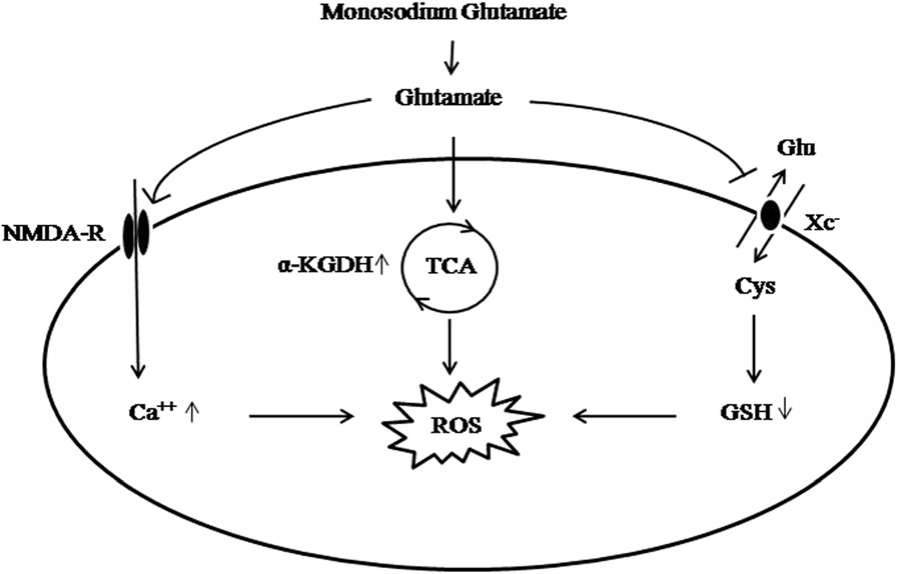
A proposed model of MSG-induced ROS production in the rat kidney. Glutamate upon chronic MSG exposure may raise the activity of α-ketoglutarate dehydrogenase, a potential ROS generator. Additionally, an increased intracellular calcium level via glutamate receptors can stimulate free radical generation and lipid peroxidation. Inhibition of cystine uptake leads to decreased GSH levels that may further promote ROS-mediated renal cell damage

The possible mechanisms of MSG-induced ROS production in the kidney are illustrated in Fig. 2. ROS arises as a by-product of aerobic metabolism [45]. The main sites of ROS production are the mitochondrial electron transport system, peroxisomal fatty acid, cytochrome P-450, and phagocytic cells [46, 47]. One study suggested that the mitochondrial electron transport chain is a major source of ROS in oxidative glutamate toxicity [48] and that extracellular glutamate level increases the formation of hydroxyl radicals [49]. Most cellular ROS arise due to leakage of electrons from the mitochondrial respiratory chain. In normal physiological conditions, ROS produced as a byproduct of metabolic processes are completely inactivated by cellular and extracellular defense mechanisms. Nutrient metabolism can affect the production of oxidative stress in the kidney by altering energy metabolism. In this scenario, α-ketoglutarate dehydrogenase (α -KGDH) is the primary site of the control of the metabolic flux through the Krebs cycle [50].

### α-Ketoglutarate dehydrogenase: an ROS generator

A recent study has shown that increased activity of α-KGDH is related to the glutamate-stimulated ROS production in rat kidneys [5]. According to this study, glutamate contributes fuel to the Krebs cycle and modulates the redox state of the cell. High glutamate concentration may increase the mitochondrial proton gradient as a result of the over production of the electron donor by the Krebs cycle, which may in turn increase the production of mitochondrial superoxide. This proposed mechanism is supported by evidence from brain tissues where α-KGDH is a potential site of ROS generation against glutamate [21]. The E3 subunit (lipoamide dehydrogenase) of α-KGDH can activate oxygen, resulting in the production of superoxide and/or hydrogen peroxide [51–53].

α-KGDH is a key and arguably the rate-limiting enzyme in the Krebs cycle. The enzyme is inhibited by its own product, succinyl-CoA, or by a high NADH/NAD^+^ ratio, as well as by a high dihydrolipoate/lipoate ratio, thereby playing an important role in cellular redox regulation [52, 54]. However, an increased level of succinyl CoA ligase in the MSG-treated kidney tissue [5] may favor the activation of α-KGDH by consuming succinyl CoA, an inhibitor. In addition, during the oxidative stress a segment of the Krebs cycle is maintained by glutamate through α-ketoglutarate [55]. Increased levels of glyceraldehyde-3-phosphate dehydrogenase as reported in MSG treated kidney [5] can also cause oxidative stress because isolated glyceraldehyde-3-phosphate dehydrogenase has been shown to catalyze NADH-dependent superoxide production [56]. Notably, NADH is one of the regulators for the activity of α-KGDH. It is possible that the excessive metabolism of glutamate in the kidney wards off the barriers to α-KGDH and thus changes the redox state of the cell. Further studies exploring the relationship between energy metabolism and oxidative stress in MSG-treated kidneys are necessary to elucidate this phenomenon.

### Glutamate receptors

Most studies in the literature link oxidative stress and tissue damage through glutamate receptor (N-methyl –D- aspartate, NMDA) via calcium (Ca^2+^) in MSG-induced renal toxicity. There are two categories of receptors available to glutamate: ionotropic and metabotropic receptors [57]. Nearly all of the known glutamate receptors and many of their interacting proteins have been detected in the kidney [58–60]. Most of the functional studies of the kidney have examined NMDA receptors, a subtype of ionotropic receptor, and group 1 metabotropic glutamate receptors (mGluRs).

NMDA receptors are Ca^2+^ favoring glutamate gated ion channels, whereas mGluRs are coupled to G protein cascades [19, 61]. The functional significance of these receptors for normal kidney physiology is not well understood. But, increased NMDA receptor subunit NR1 and NR2C expression correlates with the renal damage in a rat model of gentamicin nephrotoxicity [62]. Furthermore, a study applying NMDA receptor agonists (glycine, glutamate) and antagonists (MK 801, CPP) in renal culture cells has demonstrated that an excessive stimulation or blockade of the renal NMDA receptor results in cell death [12]. Sustained activation of these receptors induces changes in cellular Ca^2+^ dynamics that can trigger numerous cellular reactions, including the activation of nitric oxide synthase and protein kinase C [63, 64]. These in turn can activate free radical generation and lipid peroxidation [65], leading to cell damage. This mechanism of excitotoxicity has been described not only in neurons but also in lung [19, 64]. However, there is no direct evidence in the literature of studies investigating the role of glutamate receptors against MSG-induced renal cell damage; experiments with the blockade of NMDA receptor to prevent MSG-induced toxicity could be conclusive.

### Cystine-glutamate antiporter

The cystine-glutamate antiporter, designated as system x_c_^-^, exchanges extracellular cystine for intracellular glutamate in a variety of cells [66]. The uptake of cystine that results from cystine-glutamate exchange is critical in maintaining the levels of glutathione, a critical antioxidant [67]. Under the condition of oxidative stress, the transport activity of this carrier appears to be up-regulated [68, 69].

Considering the fact that the system x_c_^-^ is strongly expressed in the kidney [70] and the decreased GSH levels are prominent in MSG-induced renal toxicity, our group investigated the expression level of system x_c_^-^ in acute and chronic MSG-treated kidney. However, no significant changes were observed at the mRNA level (unpublished data). Notably, there are other minor transporters for cystine intake into the cell as well. In another study, marked inhibition of cystine uptake by glutamate in the five-day-cultured renal tubule cells of rats but not in uncultured cells has been observed [71]. Despite these findings, more studies are necessary to find the possible involvement of cystine-glutamate antiporter in MSG-induced oxidative kidney damage. It is important to note here that glutamate toxicity in the neuronal cells involves the inhibition of system x_c_^-^, leading to oxidative stress [20].

## Conclusions

During the last decade it became apparent that the chronic intake of MSG has potential effects on the peripheral organs such as the kidneys. Reduced antioxidant enzymes, increased lipid peroxidation, and tubulo-interstitial fibrosis brought on by high MSG intake strongly support the theory that oxidative stress is central to MSG-induced renal toxicity, with α-KGDH as a key player. Also, there is now evidence that excessive NMDA receptor activation is toxic for renal cells. However, a more clear association has to be established between α-KGDH, glutamate receptors, cystine-glutamate antiporter, and chronic MSG intake in order to provide a more comprehensive mechanism of renal oxidative stress. Approaches utilizing high throughput in vitro methods are crucial.

## Abbreviations

GSH: Glutathione
HNE: Hydroxynoneal
MDA: Malondialdehyde
MSG: Monosodium glutamate
NAC: N-acetylcysteine
NAD^+^: Nicotinamide adenine dinucleotide
NMDA: N-methyl -D- aspartate
ROS: Reactive oxygen species
α-KGDH: α-Ketoglutarate dehydrogenase

## References

[1] Diniz YS, Fernandes AA, Campos KE, Mani F, Ribas BO, Novelli EL (2004). Toxicity of hypercaloric diet and monosodium glutamate: oxidative stress and metabolic shifting in hepatic tissue. Food Chem Toxicol.

[2] Farombi EO, Onyema OO (2006). Monosodium glutamate-induced oxidative damage and genotoxicity in the rat: modulatory role of vitamin C, vitamin E and quercetin. Hum Exp Toxicol.

[3] Pavlovic V, Pavlovic D, Kocic G, Sokolovic D, Sarac M, Jovic Z (2009). Ascorbic acid modulates monosodium glutamate induced cytotoxicity in rat thymus. Bratisl Lek Listy.

[4] Sharma A, Prasongwattana V, Cha'on U, Selmi C, Hipkaeo W, Boonnate P (2013). Monosodium glutamate (MSG) consumption is associated with urolithiasis and urinary tract obstruction in rats. PLoS One.

[5] Sharma A, Wongkham C, Prasongwattana V, Boonnate P, Thanan R, Reungjui S (2014). Proteomic analysis of kidney in rats chronically exposed to monosodium glutamate. PLoS One.

[6] Bashan N, Kovsan J, Kachko I, Ovadia H, Rudich A (2009). Positive and negative regulation of insulin signaling by reactive oxygen and nitrogen species. Physiol Rev.

[7] Corda S, Laplace C, Vicaut E, Duranteau J (2001). Rapid reactive oxygen species production by mitochondria in endothelial cells exposed to tumor necrosis factor-alpha is mediated by ceramide. Am J Respir Cell Mol Biol.

[8] Stankiewicz A, Skrzydlewska E, Makiela M (2002). Effects of amifostine on liver oxidative stress caused by cyclophosphamide administration to rats. Drug Metabol Drug Interact.

[9] Sundaresan M, Yu ZX, Ferrans VJ, Irani K, Finkel T (1995). Requirement for generation of H2O2 for platelet-derived growth factor signal transduction. Science.

[10] Paul MV, Abhilash M, Varghese MV, Alex M, Nair RH (2012). Protective effects of alpha-tocopherol against oxidative stress related to nephrotoxicity by monosodium glutamate in rats. Toxicol Mech Methods.

[11] Thomas M, Sujatha KS, George S (2009). Protective effect of Piper longum Linn. on monosodium glutamate induced oxidative stress in rats. Indian J Exp Biol.

[12] Leung JC, Ragland N, Marphis T, Silverstein DM (2008). NMDA agonists and antagonists induce renal culture cell toxicity. Med Chem.

[13] Kubo K, Saito M, Tadokoro T, Maekawa A (1997). Changes in susceptibility of tissues to lipid peroxidation after ingestion of various levels of docosahexaenoic acid and vitamin E. Br J Nutr.

[14] Richter C, Park JW, Ames BN (1988). Normal oxidative damage to mitochondrial and nuclear DNA is extensive. Proc Natl Acad Sci U S A.

[15] Rubbo H, Radi R, Trujillo M, Telleri R, Kalyanaraman B, Barnes S (1994). Nitric oxide regulation of superoxide and peroxynitrite-dependent lipid peroxidation. Formation of novel nitrogen-containing oxidized lipid derivatives. J Biol Chem.

[16] Stadtman ER, Levine RL (2000). Protein oxidation. Ann N Y Acad Sci.

[17] Klahr S (1997). Oxygen radicals and renal diseases. Miner Electrolyte Metab.

[18] Vielhauer V, Anders HJ, Mack M, Cihak J, Strutz F, Stangassinger M (2001). Obstructive nephropathy in the mouse: progressive fibrosis correlates with tubulointerstitial chemokine expression and accumulation of CC chemokine receptor 2- and 5-positive leukocytes. J Am Soc Nephrol.

[19] Jahr CE, Stevens CF (1993). Calcium permeability of the N-methyl-D-aspartate receptor channel in hippocampal neurons in culture. Proc Natl Acad Sci U S A.

[20] Murphy TH, Miyamoto M, Sastre A, Schnaar RL, Coyle JT (1989). Glutamate toxicity in a neuronal cell line involves inhibition of cystine transport leading to oxidative stress. Neuron.

[21] Zundorf G, Kahlert S, Bunik VI, Reiser G (2009). alpha-Ketoglutarate dehydrogenase contributes to production of reactive oxygen species in glutamate-stimulated hippocampal neurons in situ. Neuroscience.

[22] Pfaller W, Gstraunthaler G, Willinger CC (1990). Morphology of renal tubular damage from nephrotoxins. Toxicol Lett.

[23] Dixit SG, Rani P, Anand A, Khatri K, Chauhan R, Bharihoke V (2014). To study the effect of monosodium glutamate on histomorphometry of cortex of kidney in adult albino rats. Ren Fail.

[24] Ortiz GG, Bitzer-Quintero OK, Zarate CB, Rodriguez-Reynoso S, Larios-Arceo F, Velazquez-Brizuela IE (2006). Monosodium glutamate-induced damage in liver and kidney: a morphological and biochemical approach. Biomed Pharmacother.

[25] Ozaki M, Yamada Y, Matoba K, Otani H, Mune M, Yukawa S (1999). Phospholipase A2 activity in ox-LDL-stimulated mesangial cells and modulation by alpha-tocopherol. Kidney Int Suppl.

[26] Bowie AG, O'Neill LA (2000). Vitamin C inhibits NF-kappa B activation by TNF via the activation of p38 mitogen-activated protein kinase. J Immunol.

[27] Massy ZA, Guijarro C, O'Donnell MP, Kim Y, Kashtan CE, Egido J (1999). The central role of nuclear factor-kappa B in mesangial cell activation. Kidney Int Suppl.

[28] Han D, Sen CK, Roy S, Kobayashi MS, Tritschler HJ, Packer L (1997). Protection against glutamate-induced cytotoxicity in C6 glial cells by thiol antioxidants. Am J Physiol.

[29] Penugonda S, Ercal N (2011). Comparative evaluation of N-acetylcysteine (NAC) and N-acetylcysteine amide (NACA) on glutamate and lead-induced toxicity in CD-1 mice. Toxicol Lett.

[30] Ribeiro G, Roehrs M, Bairros A, Moro A, Charao M, Araujo F (2011). N-acetylcysteine on oxidative damage in diabetic rats. Drug Chem Toxicol.

[31] Ye J, Li J, Yu Y, Wei Q, Deng W, Yu L (2010). L-carnitine attenuates oxidant injury in HK-2 cells via ROS-mitochondria pathway. Regul Pept.

[32] Cimolai MC, Vanasco V, Marchini T, Magnani ND, Evelson P, Alvarez S (2014). alpha-Lipoic acid protects kidney from oxidative stress and mitochondrial dysfunction associated to inflammatory conditions. Food Function.

[33] Hamza RZ, Al-Harbi MS (2014). Monosodium glutamate induced testicular toxicity and the possible ameliorative role of vitamine E or selenium in male rats. Toxicol Rep.

[34] de Groot AP, Feron VJ, Immel HR (1988). Induction of hyperplasia in the bladder epithelium of rats by a dietary excess of acid or base: implications for toxicity/carcinogenicity testing. Food Chem Toxicol.

[35] Stegink LD, Brummel MC, Boaz DP, Filer LJ (1973). Monosodium glutamate metabolism in the neonatal pig: conversion of administered glutamate into other metabolites in vivo. J Nutr.

[36] Vercoutere B, Durozard D, Baverel G, Martin G (2004). Complexity of glutamine metabolism in kidney tubules from fed and fasted rats. Biochem J.

[37] Hediger MA (1999). Glutamate transporters in kidney and brain. Am J Physiol.

[38] Vinay P, Lemieux G, Gougoux A, Halperin M (1986). Regulation of glutamine metabolism in dog kidney in vivo. Kidney Int.

[39] Khan SR, Glenton PA, Backov R, Talham DR (2002). Presence of lipids in urine, crystals and stones: implications for the formation of kidney stones. Kidney Int.

[40] Talham DR, Backov R, Benitez IO, Sharbaugh DM, Whipps S, Khan SR (2006). Role of lipids in urinary stones: studies of calcium oxalate precipitation at phospholipid langmuir monolayers. Langmuir.

[41] Ricardo SD, Diamond JR (1998). The role of macrophages and reactive oxygen species in experimental hydronephrosis. Semin Nephrol.

[42] Yeh CH, Chiang HS, Lai TY, Chien CT (2011). Unilateral ureteral obstruction evokes renal tubular apoptosis via the enhanced oxidative stress and endoplasmic reticulum stress in the rat. Neurourol Urodyn.

[43] Sampson N, Koziel R, Zenzmaier C, Bubendorf L, Plas E, Jansen-Durr P (2011). ROS signaling by NOX4 drives fibroblast-to-myofibroblast differentiation in the diseased prostatic stroma. Mol Endocrinol.

[44] Barnes JL, Gorin Y (2011). Myofibroblast differentiation during fibrosis: role of NAD(P)H oxidases. Kidney Int.

[45] Coyle JT, Puttfarcken P (1993). Oxidative stress, glutamate, and neurodegenerative disorders. Science.

[46] Aguilaniu H, Gustafsson L, Rigoulet M, Nystrom T (2003). Asymmetric inheritance of oxidatively damaged proteins during cytokinesis. Science.

[47] Beckman KB, Ames BN (1998). The free radical theory of aging matures. Physiol Rev.

[48] Tan S, Sagara Y, Liu Y, Maher P, Schubert D (1998). The regulation of reactive oxygen species production during programmed cell death. J Cell Biol.

[49] Yang CS, Tsai PJ, Lin NN, Liu L, Kuo JS (1995). Elevated extracellular glutamate levels increased the formation of hydroxyl radical in the striatum of anesthetized rat. Free Radic Biol Med.

[50] Hansford RG (1980). Control of mitochondrial substrate oxidation. Curr Top Bioenerg.

[51] Massey V (1994). Activation of molecular oxygen by flavins and flavoproteins. J Biol Chem.

[52] Starkov AA, Fiskum G, Chinopoulos C, Lorenzo BJ, Browne SE, Patel MS (2004). Mitochondrial alpha-ketoglutarate dehydrogenase complex generates reactive oxygen species. J Neurosci.

[53] Tretter L, Adam-Vizi V (2005). Alpha-ketoglutarate dehydrogenase: a target and generator of oxidative stress. Philos Trans R Soc Lond B Biol Sci.

[54] Bunik VI (2003). 2-Oxo acid dehydrogenase complexes in redox regulation. Eur J Biochem.

[55] Yudkoff M, Nelson D, Daikhin Y, Erecinska M (1994). Tricarboxylic acid cycle in rat brain synaptosomes. Fluxes and interactions with aspartate aminotransferase and malate/aspartate shuttle. J Biol Chem.

[56] Chan PC, Bielski BH (1980). Glyceraldehyde-3-phosphate dehydrogenase-catalyzed chain oxidation of reduced nicotinamide adenine dinucleotide by perhydroxyl radicals. J Biol Chem.

[57] Willard SS, Koochekpour S (2013). Glutamate, glutamate receptors, and downstream signaling pathways. Int J Biol Sci.

[58] Gu L, Liang X, Wang L, Yan Y, Ni Z, Dai H (2012). Functional metabotropic glutamate receptors 1 and 5 are expressed in murine podocytes. Kidney Int.

[59] Puliti A, Rossi PI, Caridi G, Corbelli A, Ikehata M, Armelloni S (2011). Albuminuria and glomerular damage in mice lacking the metabotropic glutamate receptor 1. Am J Pathol.

[60] Rastaldi MP, Armelloni S, Berra S, Calvaresi N, Corbelli A, Giardino LA (2006). Glomerular podocytes contain neuron-like functional synaptic vesicles. FASEB J.

[61] Aramori I, Nakanishi S (1992). Signal transduction and pharmacological characteristics of a metabotropic glutamate receptor, mGluR1, in transfected CHO cells. Neuron.

[62] Leung JC, Marphis T, Craver RD, Silverstein DM (2004). Altered NMDA receptor expression in renal toxicity: Protection with a receptor antagonist. Kidney Int.

[63] Lan JY, Skeberdis VA, Jover T, Grooms SY, Lin Y, Araneda RC (2001). Protein kinase C modulates NMDA receptor trafficking and gating. Nat Neurosci.

[64] Said SI, Berisha HI, Pakbaz H (1996). Excitotoxicity in the lung: N-methyl-D-aspartate-induced, nitric oxide-dependent, pulmonary edema is attenuated by vasoactive intestinal peptide and by inhibitors of poly(ADP-ribose) polymerase. Proc Natl Acad Sci U S A.

[65] Babu GN, Bawari M, Ali MM (1994). Lipid peroxidation potential and antioxidant status of circumventricular organs of rat brain following neonatal monosodium glutamate. Neurotoxicology.

[66] Bridges R, Lutgen V, Lobner D, Baker DA (2012). Thinking outside the cleft to understand synaptic activity: contribution of the cystine-glutamate antiporter (System xc-) to normal and pathological glutamatergic signaling. Pharmacol Rev.

[67] Orrenius S, Ormstad K, Thor H, Jewell SA (1983). Turnover and functions of glutathione studied with isolated hepatic and renal cells. Fed Proc.

[68] Kim JY, Kanai Y, Chairoungdua A, Cha SH, Matsuo H, Kim DK (2001). Human cystine/glutamate transporter: cDNA cloning and upregulation by oxidative stress in glioma cells. Biochim Biophys Acta.

[69] Miura K, Ishii T, Sugita Y, Bannai S (1992). Cystine uptake and glutathione level in endothelial cells exposed to oxidative stress. Am J Physiol.

[70] Burdo J, Dargusch R, Schubert D (2006). Distribution of the cystine/glutamate antiporter system xc- in the brain, kidney, and duodenum. J Histochem Cytochem.

[71] Foreman JW, McNamara PD, Bowring MA, Lee J, Rea C, Segal S (1986). Cystine-glutamate transport interactions in rat renal cortical tubules, brushborder vesicles, and cultured renal tubule cells. Biosci Rep.

